# Hidden work at the fissured workplace: ambivalent visibility in daytime cleaning

**DOI:** 10.3389/fsoc.2026.1696463

**Published:** 2026-02-05

**Authors:** Karin Sardadvar

**Affiliations:** Department of Education, University of Vienna, Vienna, Austria

**Keywords:** cleaning, daytime cleaning, fissured workplace, hidden work, invisible work, outsourcing, service triangle

## Abstract

Cleaning work has been subject to outsourcing since the 1980s, leading to what David Weil has termed *fissured workplaces*. In such settings, cleaners’ work takes place in the complex *service triangle* of employer, employee, and client. Due to outsourcing and to low appreciation, cleaning work, much of which is done by many female and migrant workers, often takes place as *hidden work* or *invisible work*. As I argue in this paper, one aspect that renders cleaning work invisible is the working times, a dynamic that I call *temporal invisibility*. Cleaning often takes place at the margins of the day and with fragmented working times. An alternative is *daytime cleaning*, where cleaners and employees of the customer company work at the same time. In this paper, I investigate what happens to cleaners’ (in)visibility in daytime cleaning settings. Based on qualitative research in the Austrian cleaning sector including interviews and participant observation, I show that cleaners’ (in)visibility is complex and ambivalent. The findings demonstrate that in the service triangle, employees of the customer company can exert a large influence on cleaners’ working conditions—in spite of not being formally in charge. They do so by monitoring cleaners, imposing their own working times on cleaners’ working routines, and by reproducing inequality in everyday interaction. Furthermore, the findings also show which strategies cleaners use to render themselves more or less invisible. I conclude that contradictions need to be taken into account in the concept of (in)visibility, and that the introduction of daytime cleaning requires accompanying measures that also address the employees of the customer company. To improve cleaners’ working conditions in contexts of the service triangle and the fissured workplace, I argue that measures can be taken on different levels at the same time.

## 1 Introduction

A company’s employees are not the only people who work for the firm: In fact, many organizations employ additional staff via outsourcing, contracting-out, or subcontracting. While these people do not formally belong to the organization, they often work on its premises, sometimes even in interaction with the organization’s employees, and they contribute to the organization’s running and success. As Weil (2014) argues in his concept of the *fissured workplace*, companies are also responsible for those workers whom they employ in indirect ways—but this responsibility is often neglected.

In this contribution, I draw on the concept of the *fissured workplace* (Weil, 2014) and link it to concepts of *hidden work* and *invisible work* (Crain et al., 2016; Hatton, 2017; Noon and Blyton, 2002) to investigate the empirical case of outsourced cleaning work with a focus on office cleaning. Cleaners doing outsourced cleaning work usually work at the customers’ premises. Yet, they are mostly not integrated into the customer company, and sometimes even openly ignored or devalued (Costas, 2022; Sardadvar and Reiter, 2024). On the one hand, I argue, it is the way that their working times are organized that contributes to their invisibility: Often working at the margins of the day, the cleaners and their work are hidden from other building users. On the other hand, in cases in which cleaners and other building users do work at the same time, my research suggests that their interactions reflect the social inequalities their relation is structurally built on.

This study is based on qualitative empirical data from the Austrian cleaning sector, including interviews and participant observation. The data was collected in such a way as to allow for comparisons between cleaning work that is hidden by being moved to atypical working times and daytime cleaning settings where cleaners and other building users work at the same time. The findings show that working in daytime cleaning can reduce cleaners’ isolation at work and improve their quality of life as a whole. At the same time, the findings reveal subtle everyday devaluations that mirror structural inequalities linked to the cleaners’ position at the customer companies and their overall social status. These novel findings add to previous evidence for the ambivalence of invisibility, and for the reproduction of inequalities in everyday cleaning work settings [see von Bose (2017), Costas (2022), Rabelo and Mahalingam (2019), Sardadvar (2016), and Sardadvar et al. (2015)], while centering the links between working times and invisibility.

In this paper, I investigate the question of what happens to cleaners’ visibility and appreciation if outsourced cleaning is conducted during the usual office hours. I analyze how their work is rendered (in)visible in the service triangle of the fissured workplace, and what this implies for cleaners’ acknowledgement. The findings show that on the one hand, daytime cleaning has strong potential to improve cleaners’ experiences of work and their lives. On the other hand, the visibility linked to daytime cleaning proves to be contradictory. As such, this study expands existing research on the topic of (in)visibility and social inequality in cleaning work, linking it to concepts of hidden and invisible work, and to the structural contexts of workplace fissuring and service triangles.

The paper is organized as follows: I start with the theoretical concepts framing the study. Firstly, I describe the concept of the fissured workplace and the setting of the service triangle. Secondly, I discuss concepts of hidden or invisible work, as well as the proposal to include working times, i.e., *temporal invisibility*, in existing concepts. Subsequently, I present the methods used and the data collected for this study. In the findings section, I show how employees expect cleaners to work ‘invisibly’, how cleaners organize their work accordingly, how interactions between cleaners and customer company employees take place, and how it can be either helpful or not for cleaners to be seen. I close with a discussion of conceptual and institutional conclusions.

### 1.1 The fissured workplace and the service triangle

Weil’s (2014) concept of the *fissured workplace* offers an analytical framework for understanding contemporary transformations in employment relations that is highly relevant for outsourced service work such as cleaning. The fissured workplace implies a structural reorganization of economic responsibility within capitalist production. Rather than maintaining vertically integrated employment models, large corporations—which Weil calls lead businesses—increasingly focus on their most profitable functions and delegate peripheral tasks to external firms. Weil (2014) emphasizes that fissuring employment is not reducible to traditional outsourcing or subcontracting alone. It reflects a deeper logic of restructuring organizations. In a fragmented structure, formal responsibility for wages and working conditions is transferred downward, while strategic control—via branding, contractual enforcement, and performance monitoring—remains with the lead firm.

The cleaning industry represents a paradigmatic site of this reorganization, a process that started to accelerate in the 1980s. Janitorial labor, once commonly performed by directly employed staff, was among the first tasks to be outsourced (Weil, 2014, pp. 90–91). Office cleaning, in the focus of this paper, soon followed. The result is a dense landscape of large facility management companies, medium-size cleaning subcontractors, and small-scale service providers, generally competing under intense price pressure and relying on often low-skilled, partly precarious, frequently immigrant and mainly female labor (Weil, 2014, p. 133; for empirical examples, see Holtgrewe et al., 2015). Empirical studies cited by Weil (2014, pp. 132–133) show that outsourced janitors earn significantly less and are more exposed to violations such as unpaid work hours and lack of benefits. Yet, the problem is systemic, not simply a matter of misconduct by individual firms.

Indeed, looking more closely at cleaning work, several studies in the international context have suggested that the outsourcing of cleaning since the 1980s has led to the deterioration of working conditions for cleaners. Existing research has reported heavier workloads, a higher pace of work, heightened psychological pressure, and fragmented or quickly changing work schedules (Aguiar and Herod, 2006; Gather et al., 2005; Krzeslo et al., 2014). For trade unions, this is linked to difficulties in reaching the increasingly fragmented, mobile, and diverse workforces (Dingeldey et al., 2015; Larsen et al. 2019, 14 f.; Soni-Sinha and Yates, 2013). The cleaning sector exemplifies how fissured employment creates a decoupling between authority and accountability. Lead firms maintain de facto control over output quality and brand standards, yet disavow responsibility for labor outcomes (Weil, 2014, p. 121). This institutionalized ambiguity exacerbates precarity and challenges existing regulatory frameworks that still assume a clear, direct employer-employee relationship (Weil, 2014, p. 214).

Meanwhile, service sector research has identified the *service triangle*—encompassing the employer, client, and employee—as a crucial context shaping the working conditions in outsourced service work (Korczynski, 2002; Leidner, 1996). In such constellations, the client is strongly involved in shaping the working conditions. Flexibility is used to adapt the working times as closely as possible to the demands or necessities of clients (Hermann et al., 2016, p. 60). Moreover, many service workers, such as outsourced cleaners, do not perform their everyday work at their employers’ premises, but at the clients’, which means that they work *for* one company but *at* another one. In this context, Supiot (2001, p. 20f.) states:

Under legal sub-contracting, there is in principle no legal relationship between one company and the employees of another. And yet the employees’ lot may depend more on decisions made by the principal than on those of their actual employer.

As will be shown in the findings section, this has implications for cleaning workers’ social integration at work but also for uncertainties with regard to hierarchies and control.

In addition, the cleaning sector is a case in point for intersecting social inequalities and discrimination. In many countries the workforce in the cleaning sector is dominated by women (Aguiar and Herod, 2006; EFCI, 2017; Jarrow Insights, 2023). At the same time, cleaning work is typically also segmented by gender *within* the sector (Sardadvar et al., 2015; Sardadvar, 2016; Schürmann, 2013). Gender-biased interpretations of the hardships of the work are reflected in gendered job classifications in collective agreements and result in gendered employment conditions and pay gaps (Coyle, 1985; Gather et al., 2005; Rowbotham, 2006; Sardadvar et al., 2015). Furthermore, migrants have been shown to be structurally placed in more precarious parts of the sector, and to experience discrimination and racism (Baatz and Schroth, 2006; Costas, 2022; Gather et al., 2005; Santos et al., 2023; Soni-Sinha and Yates, 2013). Inequalities and discrimination based on gender in this sector strongly intersect with ethnicity and class, making cleaners a particularly vulnerable group of workers (Abbasian and Hellgren, 2012; Orupabo and Nadim, 2019; Santos et al., 2023). As such, cleaning is devalued as feminized work and reflects social and racialized inequalities in society (Costas, 2022; Sardadvar, 2016; Schürmann, 2013). While the cleaning sector is thus a case in point for a fissured workplace in the sense of Weil (2014) and an arena of (re)producing inequalities, I argue in the following that its status is further lowered by ways in which the work in the sector is made invisible.

### 1.2 Invisible work and working times

The concepts of *invisible work* or *hidden work* emerged as a response to dominant understandings of labor that privilege visibility, formalization, and monetary compensation. Daniels (1987) emphasizes how unpaid domestic labor, typically carried out by women, is systematically devalued and excluded from the societal definition of ‘real’ work. Daniels identifies three elements shaping this exclusion: the public-private divide, the presence or absence of financial compensation, and the gendered perception of what constitutes work. Yet, hidden or invisible work—I use the two terms as synonyms—also takes place in paid settings.

Noon and Blyton (2002, p. 187) conceptualize *hidden work* in contrast to formal or visible work—defined as sectors where the goods and services produced are included in official statistics, where the workers receive a wage, and where this wage is subject to tax. They mention two main forms of hidden work: firstly, concealed work, which is actively kept out of sight because it involves illegal or stigmatized activities (e.g., deviant or non-declared work). Secondly, they refer to unrecognized work, referring primarily to unpaid domestic work and voluntary work.

Hatton (2017) further elaborates the concept of invisible work, making it suitable for analyzing formal employment settings as well. In her approach, she suggests looking at three intersecting mechanisms that make work invisible: socio-cultural, socio-legal, and socio-spatial mechanisms of invisibility. The first category, ‘socio-cultural mechanisms’, refers to ideologies of gender, race, class, age, sexuality, ability, and more (Hatton, 2017, p. 338 f.). These can operate on the level of workers’ bodies (hidden bodily labor) or on the level of workers’ skills (naturalization and devaluation of skills). As I have analyzed elsewhere with regard to cleaning (Sardadvar, 2022), there is a gendered devaluation and naturalization of cleaning as a ‘female’ or ‘housewife’ skill. Hatton’s second category, ‘socio-legal mechanisms’, refers to making work invisible by excluding it from legal definitions of employment (Hatton, 2017, p. 341). Hatton mentions, among other examples, unpaid interns, working prisoners, and workshops for people with disabilities. Regarding cleaning work, this dimension can be applied to some contexts of unpaid or informally paid domestic work (Sardadvar, 2022).

The third category, ‘socio-spatial mechanisms’, is the one that applies most clearly to the problem of invisibility in commercial cleaning work, which is the focus of this paper. Here Hatton includes work that is devalued because it is performed in the domestic sphere or at other non-traditional worksites (Hatton, 2017, p. 343). Yet, I have argued (Sardadvar, 2022, p. 34 f.) that it is also the working times that can render the work invisible. This refers to the working times that are typical for the commercial cleaning sector, especially in the important area of office cleaning. In many countries, office cleaners work before and after the operating hours of the customer companies in order not to disturb the employees (Aguiar and Herod, 2006; EFCI, 2017; Jarrow Insights, 2023). This renders their work invisible in the sense that the cleaners are not met physically and are not seen doing their jobs (Sardadvar, 2019, 2022; see also Costas, 2022; Rabelo and Mahalingam, 2019). Hence, I suggest to consider working times, and thus temporal mechanisms, as a further aspect of Hatton’s ‘socio-spatial mechanisms’, and to regard what I call *temporal invisibility* as one aspect of how invisibility is produced.

As the working times in the sector are gendered (Sardadvar, 2016; Sardadvar et al., 2015; Schürmann, 2013), so is visibility. Duffy (2007) further stresses the importance of intersectionality, showing how moving reproductive work from private to public spheres has increasingly been racialized. Research by Santos et al. (2023, p. 11) shows how visibility in the cleaning sector can vary according to gender and ethnicity. In general, visibility—such as being known by name—was regarded as positive by the cleaners interviewed. However, visibility was found to be gendered: men were more easily excused for mistakes and their successes were more highly valued because they were perceived as tokens in this sector (Santos et al., 2023, p. 14). Such gendered differences in treating men in the cleaning sector more generously were also found by Schürmann (2013), Sardadvar (2016), and Sardadvar et al. (2015).

Altogether, working times in today’s cleaning sector tend to be fragmented and atypical (see Sardadvar and Reiter, 2024, p. 88). Employees in the cleaning industry in many regions of the world work part-time, with atypical contracts and unsociable working hours (Aguiar and Herod, 2006; Jarrow Insights, 2023). In Europe, it is common for some cleaning staff to work at the beginning and end of the day, or during the night, and to have split shifts (EFCI, 2017; Holtgrewe et al., 2015; Jarrow Insights, 2023). In split shifts, employees work two (or more) short shifts in one day, with an unpaid interruption of several hours in between. Such working hours are particularly typical for *office cleaning*, the largest area of activity within the cleaning industry in Europe (EFCI, 2017).

Working hours at the beginning and end of the day and split shifts have a variety of consequences for employees: they can be associated with isolation and insecurity on the job, restrictions on social and family life, and insufficient time for rest and relaxation [see Sardadvar and Reiter (2023)]. The impact of split shifts on childcare is ambivalent, as our current and previous research suggests (Gustavsson and Sardadvar, 2022; Holtgrewe et al., 2015). On the one hand, early morning shifts are not compatible with the opening times of childcare facilities. On the other hand, some workers report that organizing the evening shifts works smoothly with the help of partners or other relatives. Yet, this is hard to manage for single parents or parents, especially for migrants without relatives close by.

What changes, then, for workers’ visibility and appreciation, if cleaning is conducted during the usual office hours, i.e., as *daytime cleaning*, with office workers and cleaners working at the same time [for a definition of daytime cleaning, see ArbeitGestalten (2022, p. 18) and Sardadvar and Reiter (2024, p. 90)]? How, in daytime cleaning settings, are cleaners and their work rendered (in)visible in the service triangle of the fissured workplace? What does this imply for cleaners’ acknowledgement? These questions are in the focus of this paper.

## 2 Materials and methods

This study is based on empirical qualitative research in the Austrian cleaning sector conducted between 2018 and 2023 as part of a research project on fragmented working times in cleaning work. The overall project collected a variety of data on the cleaning sector, summarized in Table 1. For this paper, I focus particularly on two organizational case studies conducted on the Austrian cleaning industry, which investigated work shifts and daytime cleaning (see Table 2). The first case, ABC-CLEAN (all names are pseudonyms), is the subsidiary of an Austrian municipality and as such primarily responsible for cleaning public buildings. In this company, split shifts are the rule for full-time workers. In this organization, we conducted two qualitative interviews (Froschauer and Lueger, 2020) with cleaners and an expert interview (Bogner et al., 2014) with an area manager.

**Table 1:** Overview on cases/project parts, methods of data collection and analysis, and participants.

Case/Project part	Method of data collection	Participants	Method of analysis and additional information
CLEANWELL case study (conducted in 2022)	Participant observation	3 office cleaners, client company employees	3 participant observations during work shifts; researcher shadowing one cleaner at a time Analysis of field notes: Grounded Theory coding (Charmaz, 2014)
	1 semi-structured expert interview (duration: 65 min)	Head of cleaning division	Analysis of transcript: Grounded Theory coding (Charmaz, 2014)
	1 qualitative group interview (duration: 30 min)	3 office cleaners	Interview conducted after observations with the same cleaners at their work site Analysis of transcript and field notes: Grounded Theory coding (Charmaz, 2014)
ABC-CLEAN case study (conducted in 2021), including student’s master thesis (conducted in 2022/23)	2 semi-structured expert interviews (duration: 30–60 min)	Cleaning company manager; labor market expert	Analysis of transcripts: Grounded Theory coding (Charmaz, 2014; main study) and Thematic Analysis (Lueger, 2010; master thesis)
	Participant observation	Office cleaners	Part of master thesis Analysis of field notes with observation analysis (Lueger, 2010) (No observation during the main study due to the COVID-19 pandemic)
	8 qualitative interviews (duration: 30–60 min)	Office cleaners with and without daytime cleaning schemes	Analysis of transcripts: Grounded Theory coding (Charmaz, 2014; main study) and Thematic Analysis (Lueger, 2010; master thesis)
COVID-19 sub-study (conducted in 2021)	7 semi-structured expert interviews (duration: 30–60 min)	Employer representatives, employee representatives, cleaning company managers	Interviews were conducted by telephone due to the pandemic Analysis of transcripts: Grounded Theory coding (Charmaz, 2014)
Students’ sub-project (conducted in 2020)	4 semi-structured expert interviews, 1 cleaner interview	Labor market expert, cleaning company manager, cleaning coordinator, cleaning sector expert, cleaner	Analysis of transcripts with Thematic Analysis and System Analysis (Froschauer and Lueger, 2020)
Participatory stakeholder work (conducted in 2021)	2 events on daytime cleaning, organized with partners from Austrian ministries and social partners	Ca. 60 cleaning sector stakeholders (social partners, company managers, work councilors, social workers, trainers, labor market experts, sector experts…)	2 half-day events (one online, one on-site and partly streamed online) including thematic workshops, discussions, presentations, and ethnographic interviews; organized with partners from Austrian ministries and social partners Findings were noted as field notes and summarized in internal reports

**Table 2:** Characteristics of cleaners interviewed and observed in the main case studies.

Case	Cleaner	Characteristics	Methods of data collection
Cleanwell	Cleaner no. 1	Office cleaner, migrant, adult children, part-time, no split shifts	Group interview Participant observation
	Cleaner no. 2	Office cleaner, migrant, 3 children, part-time, no split shifts	Group interview Participant observation
	Cleaner no. 3	Office cleaner and team leader, migrant, 4 children, part-time, no split shifts	Group interview Participant observation
ABC-Clean	Cleaner no. 4	Office cleaner, born in Austria, 1 child, full-time, split shifts	Individual interview
	Cleaner no. 5	Office cleaner, migrant, 3 children, full-time, split shifts	Individual interview

The second case, CLEANWELL, is a service provider that sets comparatively high social standards and aims to offer uninterrupted work shifts during the day, i.e., daytime cleaning without split shifts. In this case, the methods used included participant observation (Spradley, 1980). Compared to interviews, which offer insights into people’s interpretations, observation can focus on actions and interactions. Three cleaners, who worked in daytime cleaning settings without split shifts, were accompanied by a researcher in their daily work. During the observations, employees of the customer company who were present were briefly interviewed by means of ethnographic interviews (Spradley, 1980). In addition, a final group interview (Froschauer and Lueger, 2020) with the three cleaners who had taken part in the observation was conducted. Group interviews (Froschauer and Lueger, 2020) allow researchers to investigate the group dynamics and structures of communication pertaining to a topic. Moreover, one expert interview (Bogner et al., 2014) with a manager was conducted.

The participant observations were conducted by an experienced researcher from the research team. The researcher remained in the background and followed the cleaners on their usual routines, talking to them without interfering in their work tasks. The client company had agreed to participate and a field visit for conducting both the participant observations and the interviews at CLEANWELL was organized jointly by CLEANWELL and the researchers. The field notes addressed both the participant observations and the context of the group interview. The field notes were included in the analysis. Both the researcher who had been on-site and a researcher who had not participated in data collection analyzed the data in order to have multiple perspectives and to enhance the quality of the interpretation. Participant observation had also been planned at ABC-CLEAN but had to be canceled due to the COVID-19 pandemic.

In addition to the organizational case studies, several additional data sources, which have also informed the findings of this study, were used for the project as a whole (see Table 1). Firstly, during the COVID-19 pandemic, an additional investigation was conducted focusing on sector experts and stakeholders. This part of the research comprised seven semi-structured expert interviews (Bogner et al., 2014) and several ethnographic interviews (Spradley, 1980) with employer representatives, employee representatives, and cleaning company managers. Secondly, as part of a Master thesis within the framework of the main project, six additional interviews in the field of daytime cleaning were conducted at ABC-CLEAN (Miller, 2023). Thirdly, as part of the research conducted in a project-related university course, the issue of working times in Austrian cleaning was investigated in a research project by a group of students (Claußen et al., 2022). Fourthly, the research was complemented by participative work with cleaning sector stakeholders in Austria. In the context of several activities (events, presentations, workshops) on the cleaning sector, interviews, ethnographic interviews, and observations were conducted and findings of joint workshops with participants collected.

Most of the data (verbatim interview transcripts, protocols of ethnographic interviews and observation protocols) was analyzed using the coding procedures of the grounded theory approach developed by Charmaz (2014). To analyze the data, we used the steps proposed by constructivist grounded theory for detailed initial coding and increasingly abstract focused coding, including writing memos (Charmaz, 2014, p. 109ff., p. 138ff.). Coding distils, sorts, and synthesizes the data in an interpretive process (Charmaz, 2014, p. 4). In a first step of open coding, the data was analyzed in a broad and undirected way, while in the next steps, the analysis became increasingly comparative and abstract. The coding process provides increasingly analytic categories grounded in the data, which, at the same time, serve to identify gaps in the emerging findings and the need for further enrichment, refinement, comparison, contrast, and differentiation. The coding was open, interpretive, and analytic in character; technically, it was supported by the software nVivo. The process of building codes and categories was understood as an active process of constructing findings involving the researchers (Charmaz, 2014). For quality control, this process was supported by having the analysis done by several researchers and in interpretation groups. Methodological rigor was further supported by the writing of memos which reflected how findings were constructed and which contextual knowledge was involved. As an example, Table 3 illustrates the category ‘producing visibility and invisibility’ and its codes.

**Table 3:** Category ‘Producing visibility and invisibility’ and some of its codes, in comparison between the two main case studies and between non-daytime and daytime cleaning settings.

Codes	ABC-CLEAN case Non-daytime cleaning	CLEANWELL case Daytime cleaning
Adapting cleaning to customer demands	Working times are often organized as split shifts and at the margins of the day; it is the cleaning company that adapts the working times to the customers’ demands.	Working times are organized as daytime cleaning; it is then the cleaners themselves who adapt their work to customer demands (e.g., by avoiding noise or squeezing noisy activities into the time window before employees arrive in the early morning).
Customers’ expectations of cleaners’ invisibility	The organization of working times at the margins of the day is linked to the expectation that cleaning should not interfere with customer employees’ work.	Invisibility is expected by some employees of the customer company (e.g., the ‘third floor’) despite daytime cleaning.
Friendly interaction between cleaners and customer employees	Little interaction (friendly or unfriendly) takes place due to working times at the margins of the day.	Friendly interactions take place that enrich cleaners’ workdays and reduce their isolation.
Unfriendly, distanced interaction	Little interaction (friendly or unfriendly) takes place due to working times at the margins of the day.	Interaction can be distanced or rude in different ways: Physical interaction without words Impolite interaction (e.g., no greeting, no “excuse me”; ignoring cleaner) Polite but distanced interaction: *cold greeting* pattern
Complaints and control	Control is exerted by the staff in charge of the outsourced facility management in the customer company, and by supervisors in the cleaning company. Contact mainly takes place between customer and employer, rarely directly addressed to cleaners.	Evaluation of the cleaners’ performance is negotiated on three levels: In relation to the employing company (by supervisors) The client company can report any deficiencies directly to the customer company There is also direct criticism from the client company’s employees to the cleaners present
Cleaners making themselves invisible	There are strongly differing individual strategies to deal with clients who ask for tasks or favors. Cleaners’ strategies can also change over time, with increased experience.	There are patterns of cleaners minimizing talking in order to avoid complaints and additional tasks required by customer company employees.
Cleaners making themselves visible	This pattern was not reported and could not be observed in this case study.	Cleaners put up ‘Caution: Wet Floor’ signs although the floor is not wet or they prop restroom doors open with waste baskets in order to keep other building users out and not to be disturbed at work.

The different parts of the research project came together as follows: First, each piece of research (case studies, Master thesis, corona sub-study etc.) was analyzed independently. Later, the findings of the different pieces of research were compared and integrated based on the evolving categories. Here are two examples of this procedure: Results from the stakeholder workshops provided insights into daytime cleaning from various perspectives. These findings on daytime cleaning were integrated with the findings on daytime cleaning of the two organizational case study findings. Also, findings from student research participant observations on how cleaners and other building users interact were compared with the observations from the main case studies, partly confirming and partly supplementing them.

In line with the EU’s General Data Protection Regulation, the Charter of Fundamental Rights of the European Union, and the Ethic Codex of the German and Austrian Sociologists (DGS and BDS, 2025), the highest ethical standards were applied during our research. All participants were informed verbally and in writing about the use of their data in the research project and their rights; they had the opportunity to ask questions and explicitly gave their informed consent. The funding institution included ethical aspects in their criteria for approval of the project.

## 3 Results

In what follows, I present findings regarding cleaners’ visibility and invisibility based on the analyzed data. First, I show how customer employees still expect cleaners to be invisible even in daytime cleaning settings, and how this, second, leads to cleaners organizing their work accordingly. Third, I discuss how interactions at the workplace can be either joyful or unpleasant. Fourth, I show that it can sometimes be advantageous for cleaners not to be seen and that they use strategies to make themselves more or less visible. Table 3 summarizes important aspects of the category ‘producing visibility and invisibility’ along the empirical codes, comparing the two case studies and the non-daytime and daytime cleaning settings based on the analysis.

### 3.1 The ‘third floor’: customers’ expectations of cleaners’ invisibility

In line with the historically grown working times at the margins of the day, employees of customer companies nowadays often expect cleaners and their work to remain invisible and, as I will show, inaudible. That is, the working times customers have become used to are the basis for ongoing expectations—and I found that they can persist even if the service is transformed to daytime cleaning.

This can be illustrated with the narration of the ‘third floor’ from the CLEANWELL case. In one customer company building, three cleaners divide up the six floors of the office building. Each cleaner is responsible for two floors. The three cleaners are generally satisfied with their working situation in the building. There is only one floor that they find unpleasant: the third floor. On this floor, the employees are not friendly and never satisfied, as the three cleaners unanimously report. The employees working in the offices of the third floor demand that the cleaners do not clean when employees of the client company are in the office. Above all, they do not want vacuuming to be done. Everything is supposed to be ready when the employees arrive in the morning. A short sequence from the group interview illustrates the cleaners’ experience with the ‘third floor’ (note that all interview quotes were translated from German to English, and that most interview partners did not speak in their first language):

Mrs. Hacioglu: They want everything ready, one hour of time, but that’s not possible.

Interviewer: Ah yes.

Mrs. Hacioglu: Everything prepared, everything nice, everything clean. It’s already ready. But where is the time?

I: Yes.

Mrs. Hacioglu: It’s a big place too.

(CLEANWELL, group interview)

The fact that the office employees expect the cleaning to be done outside their own working hours despite the daytime cleaning setting is linked to stress for the cleaners. They must hurry so that they are finished on this floor when the employees arrive. All three of them have already worked on this floor. Mrs. Machado says that she used to cry regularly because of the stress. Now Mrs. Esteban, a new employee, works on this floor. They see it as particularly problematic that the employees start working so early—some already start at 6.30 a.m. This reduces the cleaners’ time window for vacuuming. Here, we can see how the customer company employees’ own working time preferences and flexibility clash with those of the cleaners, and that they are prioritized. The case also shows that, even if they are not in a hierarchically higher position than the cleaners, the employees on-site strongly shape the cleaners’ working times and routines in the service triangle. Another sequence from the group interview shows how the cleaners experience the time pressure on the ‘third floor’:

Mrs. Esteban: But everyone comes at 6:00.

Mrs. Hacioglu: 6:30.

Mrs. Esteban: Or 6:30.

[…]

Interviewer: And what happens at 6:30? Do they say “No, you’re not allowed to clean”?

Mrs. Esteban: Yes, “Don’t clean,” but [we] still have to...

Mrs. Hacioglu: Yes. Yes, yes

Interviewer: Okay. And what do you do then, when they say: “No, no”?

Mrs. Machado: Then it’s: quickly, quickly!

Mrs. Esteban: But [we] still have to

Mrs. Hacioglu: And quickly, quickly (...) You understand, it’s stressful. Then vacuuming there, kitchen.

This example shows that in the setting of the service triangle, the employees of the client company have leeway on-site to define the working conditions of the cleaners. They refer to the visibility and, above all, audibility of the cleaners’ work, which legitimizes their claim to determine when, where, and how audibly cleaning may take place. This results in a high level of stress for the cleaners, as they have to complete their visible and audible work as quickly as possible.

This finding illustrates how in the service triangle in fissured workplaces, the immediate situation on-site becomes important despite the formal hierarchy frameworks. In settings without daytime cleaning, it is often cleaning companies that use worker flexibility in order to cater for customer demands—as in the ABC-CLEAN case, where cleaners’ working times are adapted to the client companies’ preferences. However, in the daytime cleaning case at CLEANWELL, we can see how this logic is still present when working times have been changed: The customers’ working time preferences are now directly transferred onto the cleaners to deal with in the everyday work setting on-site.

### 3.2 “Close the door, because it’s loud”: how cleaners organize their work in light of visibility and audibility

Based on customers’ expectations in the light of historically grown patterns of cleaners’ atypical working times and invisibility, cleaners organize their work so as to disturb customer company employees as little as possible and thus live up to the demand of being invisible. The participant observation as well as the ethnographic conversations and the interviews, but also the results of the participatory stakeholder work, showed that cleaners organize their work extensively so that it can be integrated into the everyday routines of the client company. As such, cleaners need a high level of organizational skills and consideration.

In the interviews and observations, it was found that cleaners strongly reflect on how perceptible or disruptive they are in their day-to-day work. One of the primary tasks is to ensure that the cleaning work interferes as little as possible with what happens on-site, or that it is as unnoticeable as possible. In the CLEANWELL case, for example, it was observed that during work that causes noise, such as handling crockery in the kitchen, cleaners adopt strategies to minimize disturbance. This can be illustrated by an episode from the participant observation with Mrs. Hacioglu:

We go back into the kitchen. Mrs. H closes the sliding door on the left and the door on the right. She puts her finger to her lips [meaning: shush, quietly] and says, ‘Close the door, because it’s loud’.

(CLEANWELL case, observation protocol 24.11., item 39)

This pattern of ‘as little disturbance as possible’ is part of the cleaners’ performance, which they implement and organize based on their experience, process knowledge and situational observations (Who is where when? What is going on and what is not going on?). Yet, this is a skill and a demand that is little acknowledged in the framing of this work which is widely regarded as unskilled.

The cleaners in the CLEANWELL case state that it is easier for them to do their work when the client company’s employees are not there. They are referring to the organization of the work, which can sometimes be like running the gauntlet when the office employees are present. For example, the cleaners find it tedious when they always have to check whether people are present and whether it is possible to clean, as the following quote shows.

I: […] And would it be better to work alone when they are not here, or is it better to work here when everyone is here?

Mrs. Machado: No, certainly when nobody is there.

I: When nobody is here?

Mrs. Machado: Yes, I’m happy in the morning. Vacuuming, yes, a bit of mopping. Because when (you) come into the kitchen, people come: ‘What’s going on? What?’ The other side, he has a lot of work. You cannot wipe down the tables or mop the floor.

(CLEANWELL, group interview)

By shifting as much cleaning work as possible to the time between 6 a.m. and 8 a.m., the pattern that cleaning work ought to be invisible is reproduced. As we can see, even in daytime cleaning settings, it is still expected that cleaning work is either simply finished when office work begins or that it is done imperceptibly. This attitude not only justifies working hours that are detrimental to cleaners’ family life and their overall well-being and does not allow for real change when daytime cleaning is introduced, but it also shifts the responsibility for remaining invisible onto the cleaners.

### 3.3 *Cold greetings*: cleaners’ workplace interaction in the service triangle

By being perceptible, daytime cleaning has an impact on the micro-processes of the client company’s employees. This can cause slight obstructions or delays, like having to push past the vacuum cleaner in the corridor, having to wait a moment in the kitchen when the cleaner is doing the dishes or having to return later when the kitchen floor has been washed. In such situations, employees interact with cleaners with their bodies or with words. We observed different forms that this interaction could take.

Sometimes, employees are polite and considerate. However, we also observed interactions in which the client company’s employees expressed their disapproval of the cleaners disturbing them. For example, we observed employees either becoming frozen or tolerating the situation in an icy manner, as well as silent or verbal requests for the cleaners to move. The following scene from an observation protocol describes such an interaction:

[In the kitchen.] Mrs. E has now gone to the doorframe. She is cleaning the light switch next to the door, blocking the doorway with her bent back and buttocks. A member of staff is carrying her filled teapot and teacup and wants to go through the door. She stands behind Mrs. E. She could not go through the door without bumping into Mrs. E. She remains standing behind Mrs. E, tense and very upright. She says ‘Excuse me?’ very quickly in a tone that is both questioning and reproachful. Mrs. E quickly straightens up and steps aside. The trashcans are there. She presses herself against the trashcans and says: ‘Excuse me, excuse me, please.’ She says this very quietly, almost in a whisper.

(CLEANWELL, observation protocol 24–11)

In these forms of reprimand, it becomes clear that the work of the cleaner, who obstructs office workers, is considered less valuable and less important than that of office employees. The cleaners’ work is not seen as an important task that requires space, but as an obstruction to the work of office employees, who are deemed more relevant. In such micro-interactions, social orders of worth, appreciation and hierarchies are reflected. This pattern ties in with the history of the fissured workplace: When companies started outsourcing services, they drew a line between key activities and other, less relevant, activities (Weil, 2014). But it is also likely to be linked to cleaning having historically become a gendered, racialized activity (Duffy, 2007). Based on this finding, I propose that for daytime cleaning to be accepted it is vital that cleaning is discursively embedded as a legitimate work activity in the client company and that expectations are mutually clarified so that daytime cleaning can fulfil its true potential.

In this context, participant observation revealed an important empirical pattern that we termed the *cold greeting*. Cold greeting refers to a form of interaction in which employees of the client organization greet cleaning staff in a cold, minimal, and emotionally distant manner. While superficially polite, these greetings serve to reinforce social distance and occupational hierarchy rather than foster interpersonal connection.

In contrast to other workplaces where cleaners may be more socially integrated—recognized by name and engaged in informal conversation—the CLEANWELL case study demonstrates a noticeable absence of such interactions, highlighting the marginalization of cleaning staff within the client’s organizational culture. According to the participant observation, communication between cleaning staff and office employees consists mainly of ‘hello’ and ‘(good) morning’. The lack of communication is also confirmed in the group interview:

Mrs. Machado: Now, no, I do not want to. Never mind, you say: Hello! Fits.

Interviewer: Just hello!

Mrs. Machado: Yes. Well, some people speak, but not everyone.

Interviewer: Yes.

Mrs. Hacioglu: Yes.

Mrs. Machado: Yes, like this, everywhere…

In the ABC-CLEAN case, in contrast, it becomes apparent that communicating with people in the client company can be very enjoyable. Mrs. Mbequo experienced the switch from split shifts to daytime cleaning, in this case at a school, as a great boost to her self-esteem. She found contact with the pupils, who soon knew her name and with whom she enjoyed chatting, to be a source of joy. This was a big difference for her compared to her previous work in split shifts, as the following interview quote shows:

Two or three children came up to me and asked me questions. And then these children left, their mother already picked them up, and she said: ‘Bye bye!’ (laughs) My God, she had already asked my name. And then she said my name and ‘Bye bye’. (laughs).

(CLEANWELL, cleaner interview, Mrs. Mbequo)

Hence, switching to daytime cleaning can make work more pleasant when cleaners become visible, known by name, and involved in friendly everyday interactions. Yet, this is not always the case, as other conversations are restricted to a few short words or to the pattern of *cold greeting* we found, which in fact creates distance. Therefore, as we will see in the following finding, cleaners themselves sometimes try to avoid being seen.

### 3.4 “All I get is a complaint”: the advantages of not being seen

While being visible is an important condition for appreciation, paradoxically, not being seen can be experienced as advantageous, too. This is the case when clients tend to control cleaners, and when they themselves prefer to avoid talking in order not to be asked to take on additional tasks or to receive complaints.

In the CLEANWELL case, the cleaner Mrs. Hacioglu says and shows that she is directly monitored by the customer company’s employees during her work. The observation protocol illustrates this:

She walks to a glass door and points to it. ‘Must check. Sometimes, definitely look for a mistake. Want to find a fault! Small spot on the glass. Then write. Must check!’ She scratches a tiny spot off the glass surface. ‘But if it’s spotless, then write!’

(CLEANWELL, observation log 24–11)

Observations reveal that cleaners think about such possible complaints and integrate them into the logic of their work processes. This means that visible dirt is prioritized for removal and exposed areas are checked particularly thoroughly. The observation shows how visible stains are always removed, even if—like on a Friday—it is not a wet mopping day:

She looks at the desk surface. She spots a stain there. She takes her microfiber cloth and wipes the stain away. Again she says: ‘Wet wipe only on Wednesday—but stain. Always check’.

(CLEANWELL, observation log 24–11)

I find that in daytime cleaning, the evaluation of the cleaners’ performance is negotiated on three levels: firstly, it is negotiated in relation to the employing company. Supervisors pay attention to compliance with agreed performance. Secondly, the client company can contact (‘write’) the employing cleaning company directly to report any deficiencies. Thirdly, there is also direct criticism from the client company’s employees to the cleaners present. The service triangle setting at the fissured workplace thus multiplies the monitoring of cleaners both on an institutional and on an interactional level.

Again, it needs to be stressed that customer company employees do not have any formal status with regard to controlling, criticizing or giving instructions. Yet, in the social setting of the service triangle, with the actual on-site presence of cleaners and customer company employees at the same time, they still do so. Unlike their employer, cleaners in daytime cleaning are immediately available and approachable for complaints. Also, we find that if the cleaners do not have enough time to complete their task, this is particularly unpleasant and stressful for them in view of the immediate assessment by customers in light of the cleaners’ own, sometimes high, quality standards and professional pride.

In this situation of being exposed to office employees’ control and critique, cleaners sometimes try to make themselves as invisible as possible. One aspect of not being seen is to minimize talking. In the CLEANWELL case, the cleaners do not necessarily want to talk to the employees. One reason for this, as they say in the group interview, is that when they are in conversation with employees and they are easy to communicate with, it is more likely for them to hear complaints, work orders, requests, etc. from the client company’s employees. It is therefore also a strategy not to get too involved in communication and not to be too approachable, as the following excerpt from the group interview shows:

Mrs. Machado: I do not know. I do not know. But anyway, just: Hello! And that’s it.

I: Just: Hello?

Mrs. Machado: When I ask something, all I get is a complaint.

(CLEANWELL, group interview)

Another case in point is when office employees ask cleaners for favors, or try to give them orders. A sequence from an observation illustrates this:

While Mrs. E cleans the microwave and kitchenette and also cleans the dishwasher, she tells me that people sometimes ask her for favors, sometimes someone comes and asks if she can do something, e.g., if drinks have been spilled on the floor, then she comes, and also if drinks have been spilled on the table.

(CLEANWELL, observation log 24–11)

In such situations, as the comparative analysis of the CLEANWELL and ABC-CLEAN cases shows, it depends on (i) the cleaners’ ability to set boundaries, (ii) the cleaners’ relationship with the employees of the client company, and (iii) the attitude of the cleaners’ supervisors to how the interaction is resolved.

In the ABC-CLEAN case, for example, Mrs. Mbequo states that she always says ‘yes’ when someone asks her to do additional work. However, she finds it burdensome because she does not really have time for such extra work. Mrs. Michleritsch from the ABC-CLEAN case, in contrast, no longer responds to additional requests. She refers to her supervisor:

Now I’ve reached the point where I tell them to call my boss if there’s a problem. So I’m really confrontational and think to myself, as harsh as it sounds: you can all get lost. If you do not like something, call the boss. So, um, it’s not entirely okay, but I do not argue anymore.

(ABC-CLEAN, cleaner interview, Mrs. Michleritsch)

The analysis suggests that the ability to set boundaries and represent oneself tends to be higher among those with good German language skills and Austrian citizenship.

Meanwhile, in order to make cleaning activities possible in everyday life, it can also be a strategy for cleaners to consciously make themselves—or, to be more precise—their activity, *visible*. When they are cleaning, certain areas are not fully available to the building users. The participant observation showed that cleaners deliberately make their work visible to indicate that they are cleaning in a certain area and that this area can therefore not be used. This can also help to avoid unpleasant negotiations. Paradoxically, thus, they make themselves visible in order to be left in peace.

For example, when Mrs. Machado cleans the kitchen or restrooms, she puts up a ‘Caution: Wet Floor’ sign. However, this sign is actually placed on a floor that does not get wet or slippery during cleaning. Instead, the sign indicates that cleaning is taking place and keeps office employees away. Mrs. Hacioglu also deliberately increases her visibility when cleaning the restrooms to draw attention to herself. She secures the waste basket for paper towels to the restroom door and uses it to hold the self-closing doors open. This makes it clear that cleaning is taking place and prevents employees from entering.

To sum up, cleaners have strategies to make themselves more or less visible and approachable. Being less visible can have advantages with regard to being monitored or receiving complaints. The service triangle setting at the fissured workplace exposes cleaners to the control and interference of customer company employees, while the employer, who is their actual superior, remains outside of the situation.

## 4 Discussion

How are the visibility and invisibility of cleaners affected when working times are changed to daytime cleaning, meaning that cleaners and other building users meet? This study shows that while daytime cleaning can improve the quality of cleaners’ work and lives, the visibility linked to it can be ambiguous. While visibility can increase attention or appreciation, it can also be hurtful. Furthermore, in some contexts, cleaners can actually have a *need for invisibility*—to do their work in peace, to not be monitored, or to avoid instructions and complaints. The findings underline and expand previous research on invisibility in cleaning work. I conclude that both interactional and structural levels need to be taken into account when investigating invisibility in cleaning work, and when designing policies aiming to improve working conditions.

In general, daytime cleaning has the potential for rendering cleaning work more visible and for enhancing cleaners’ social integration at work (Sardadvar and Reiter, 2024). Moreover, uninterrupted daytime working hours have been shown to strongly improve workers’ social, family, and private life (Sardadvar and Reiter, 2024; Sardadvar, 2024; see also Holtgrewe et al., 2015). This is underlined by research that I conducted in Norway, where daytime cleaning is relatively widespread; research which indicates that daytime cleaning improves cleaners’ work-life balance, quality of work, and their social integration within and beyond employment (Sardadvar and Reiter, 2024, and unpublished findings). Yet, in the findings presented in this paper, daytime cleaning was linked to a contradictory picture of (in)visibility. In the complicated setting of the service triangle in the fissured workplace, not being seen can in fact be of some advantage for cleaners, as I have shown, especially in order to escape from the control and demands of employees of the customer company. Moreover, while interactions with other building users can be enriching in some contexts, they can also be hurtful and reflect social inequalities between cleaners and other building users at the micro-level.

These findings tie in with, and expand, previous theoretical and empirical research on invisibility in cleaning work. Costas (2022, p. 123), who accompanied cleaners at the Potsdamer Platz in Berlin ethnographically, also found that cleaners were relieved when working in the absence of clients, feeling less monitored. As in my study, they sometimes actively made themselves invisible from clients in order not to be controlled and not to get into uncomfortable interactions. Rabelo and Mahalingam (2019) found similar patterns among university cleaners in the U.S., and von Bose (2017) among hospital cleaners in Germany. Likewise, an early study on public building custodians (Hood, 1988) showed how a mandatory change from the night to the day shift created status-management dilemmas for the custodians, as they were confronted with the building’s higher-status daytime occupants. This led to custodians’ stigmatization.

One aspect which my research adds is the paradoxical pattern of *cleaners making themselves visible in order to be able to work in peace*, for example when putting up otherwise unnecessary ‘Caution: Wet Floor’ signs. Another telling empirical pattern this research contributes is the *cold greetings* pattern: While it is known from other studies that, for cleaners, not being answered when greeting can be an intense experience of being rendered invisible (Rabelo and Mahalingam, 2019), the pattern of *cold greeting* shows that even within seemingly polite communication, social inequalities between the people involved can be reflected. Hence, it can be summarized that cleaners adopt strategies to not be visible, and thus not to be confronted with discrimination, surveillance, criticism, and extra work (see also Costas, 2022), but they also adopt hitherto less discussed strategies to be *visible*. Visibility or invisibility, I conclude, is not only a structural feature of a job or a type of work, but is also produced in individual actions and collective interactions on the micro-level of the workplace in the service triangle.

On a conceptual level, my findings support previous work which has pointed to the complexities of the invisibility concept. Visibility is not only good or bad for workers, but can be both—even at the same time. This ties in with the work of Schaffer (2015) who questions the opposition of visibility and invisibility, and stresses that the two concepts need to be seen in relation to each other. Invisibility does not always harm, sometimes it can also be empowering (von Bose, 2017; Schaffer, 2015). By the same token, visibility cannot only be linked to acknowledgement, but also to surveillance. The findings of my study underline what Costas (2022, p. 145) has summarized as follows:

All this underscores how cleaners’ invisibility is only half the problem. Invisibility implies that cleaners cannot be seen or are not noticed—that there is a lack of eyes directed toward them. Yet the opposite is equally true (Costas, 2022, p.145).

For researchers, importantly, this implies not evaluating the terms visibility and invisibility from the outset of the research, but to openly turn to the field and reconstruct (in)visibility according to the experiences of the subjects in the field and the empirical structures.

Furthermore, it is useful to differentiate between visibility *at work* and visibility *of work*, as pointed out in a study on cleaners by Rabelo and Mahalingam (2019)—and to regard both. According to this concept, invisibility *at work* is embedded in social interactions, while invisibility *of work* concerns more structural aspects—like the character of cleaning work as such, and social and vocational contexts. With regard to this differentiation, I conclude that both levels—the interactional level and the structural level—need to be taken into account when investigating invisibility in cleaning work. The same is true with regard to policies aiming to *improve* working conditions. Appropriate measures might thus be taken at different levels at the same time: For example, changing working times in order to reduce what I have called *temporal inequality* (Sardadvar and Reiter, 2024), has the potential to change a reason for invisibility at the level of organizations and industrial relations. At the same time, improving client-cleaner communication by involving client company employees in organizational change processes can be a measure to improve cleaners’ working conditions on the interactional level. Hence, *temporal invisibility*, and what von Bose (2017, 2020) calls *social invisibility*—not being seen in spite of being present [see also Rabelo and Mahalingam (2019)]—need to be addressed together.

On an organizational level, I have also shown that working at the same time as other building users may actually not be all cleaners’ first choice. On the one hand, this calls for allowing cleaners to have some say with regard to their own working times. On the other hand, this finding needs to be addressed more explicitly when daytime cleaning, otherwise very favorable for workers in many respects (Sardadvar and Reiter, 2024), is implemented. In addition, it can be concluded that it is vital on an organizational level that both cleaners and customer company employees are made aware of who the cleaners ought to report to and who not, and what the cleaning contract comprises and what it does not. Previous research on introducing daytime cleaning has emphasized that it is key to involve all parties affected by the transformation in the process (ArbeitGestalten, 2022; Miller, 2023; Sardadvar and Reiter, 2024). This is particularly important in settings of service triangles and fissured workplaces, where, as I have shown, control and surveillance of cleaners is being multiplied due to the complex arrangement of hierarchies and contact persons.

This is where the lead businesses in Weil (2014) fissured workplace concept come back into the picture: As Weil emphasizes, responsibility must be assigned to them even with regard to the employees that work for them in outsourced arrangements. Weil (2014, p. 231) suggests different measures they can adopt: For instance, lead firms can set up monitoring systems for subcontractors, they can incorporate workplace standards into the standards that are relevant for fissured workplaces, or they can set up internal training systems for affiliated organizations (Weil, 2014, p. 241). Based on the findings of this study, I add, firstly, that lead firms can involve and address their own employees with regard to the structures and changes of the fissured workplace—for example, by preparing them for a transition to daytime cleaning. As mentioned in the findings, it is vital that cleaning is discursively embedded as a legitimate work activity in the client company and that expectations are mutually clarified. Secondly, when contracting out services, working conditions can be given higher priority for choosing the subcontractor. Thirdly, the companies who contract out cleaning services could integrate responsibility for the contracted-out employees—often women, often migrants—into existing company policies for diversity management, anti-discrimination, or gender equality, but also social sustainability or corporate social responsibility if they pursue such agendas.

Yet, in light of the fissured workplace setting, my study has also highlighted a vital point for what the *employing* companies can do in order to provide good working conditions: As I have shown, in the service triangle setting, the customers’ working time preferences are directly transferred to the cleaners to deal with in the everyday work setting on-site. It is thus helpful if cleaners are able to set clear boundaries to client employees’ demands, which they can be trained to get better in. More importantly though, team leaders or property managers ought to stand behind their employees and clearly support them in situations where customers approach the cleaning staff with unagreed or inappropriate requests. In the service triangle where outsourced employees are direct exposed to the client staff, this creates an external reference structure that relieves cleaners from daily negotiations in contact with customers. To sum up, this study has provided novel empirical findings, conceptual additions, and policy-related conclusions on the topic of (in)visibility and social inequality in cleaning work. It has linked them to concepts of hidden and invisible work, as well as to the structural context of workplace fissuring and service triangles. The main limitation of the study lies in the small sample size of the organizational case studies. Comparisons along different dimensions of inequality, which previous research has suggested to be relevant—e.g., between male and female cleaners, migrant and non-migrant cleaners, or *white* cleaners and people of color [see Sardadvar (2016)]—could only be made to a limited extent. One strength of the study is that the underlying data enabled a comparison to be made between daytime cleaning and atypical working times. Such comparisons might be further strengthened in future research, which may also compare different cleaning settings (e.g., hospitals, offices, production sites) in a systematic way.

## Data Availability

The datasets presented in this article are not readily available because the qualitative data presented in this article is highly sensitive and therefore not accessible due to ethical considerations and data protection legislation. Requests to access the datasets should be directed to karin.sardadvar@univie.ac.at.
